# Multicentric phase II trial of TI‐CE high‐dose chemotherapy with therapeutic drug monitoring of carboplatin in patients with relapsed advanced germ cell tumors

**DOI:** 10.1002/cam4.3687

**Published:** 2021-03-05

**Authors:** Christine Chevreau, Christophe Massard, Aude Flechon, Rémy Delva, Gwenaëlle Gravis, Jean‐Pierre Lotz, Jacques‐Olivier Bay, Marine Gross‐Goupil, Karim Fizazi, Loïc Mourey, Angelo Paci, Jérôme Guitton, Fabienne Thomas, Bénédicte Lelièvre, Joseph Ciccolini, Sotheara Moeung, Yohan Gallois, Pascale Olivier, Stéphane Culine, Thomas Filleron, Etienne Chatelut

**Affiliations:** ^1^ Institut Claudius Regaud IUCT‐Oncopole Toulouse France; ^2^ Institut Gustave Roussy Villejuif France; ^3^ Centre Léon Bérard Lyon France; ^4^ Institut de Cancérologie de l’Ouest Centre Paul Papin Angers France; ^5^ Institut Paoli Calmettes Marseille France; ^6^ Hôpital Tenon Paris France; ^7^ CHU de Clermont‐Ferrand France; ^8^ Hôpital Saint‐André Bordeaux France; ^9^ Laboratoire de Pharmacologie Toxicologie CHU Lyon France; ^10^ Cancer Research Center of Toulouse (CRCT), Université Paul Sabatier Toulouse France; ^11^ Laboratoire de Pharmacologie Toxicologie CHU Angers France; ^12^ CHU La Timone Marseille France; ^13^ Service d'Otoneurologie et ORL Pédiatrique Hôpital Pierre Paul Riquet CHU de Toulouse Toulouse France; ^14^ Hôpital Saint‐Louis Paris France

**Keywords:** germ cell tumors, high‐dose chemotherapy, phase II trial, relapsed patients, therapeutic drug monitoring

## Abstract

**Background:**

High‐dose chemotherapy (HDCT) with TI‐CE regimen is a valid option for the treatment of relapsed advanced germ cell tumors (GCT). We report a phase II trial with therapeutic drug monitoring of carboplatin for optimizing area under the curve (AUC) of this drug.

**Methods:**

Patients with unfavorable relapsed GCT were treated according to TI‐CE regimen: two cycles combining paclitaxel and ifosfamide followed by three cycles of HD carboplatin plus etoposide administered on 3 days. Carboplatin dose was adapted on day 3 based on carboplatin clearance (CL) at day 1 in order to reach a target AUC of 24 mg.min/mL per cycle. The primary endpoint was the complete response (CR) rate.

**Results:**

Eighty‐nine patients who received HDCT were included in the modified intent‐to‐treat (mITT) analysis. Measured mean AUC was 24.4 mg.min/mL per cycle (22.4 and 26.8 mg.min/mL for 10th and 90th percentiles). Thirty‐five (44.3%) patients achieved a CR with or without surgery of residual masses and 20 patients achieved a partial response with negative tumor markers. With a median follow‐up of 44 months (m), median PFS was 12.3 m (95% CI: 7.5–25.9) and OS was 46.3 m (95% CI: 18.6–not reached). For high‐ and very high‐risk patients, according to the International Prognostic Score at first relapse or treated after at least one salvage treatment (n = 51), 2‐year PFS rate was 41.1%.

**Conclusion:**

The rates of complete and favorable responses were clinically relevant in this very poor risk population. Individual monitoring of carboplatin plasma concentration permitted to control more accurately the target AUC and avoided both underexposure and overexposure to the drug.

## Graphical abstract

The superiority of HDCT over standard chemotherapy as initial salvage treatment of patients with relapsed or refractory germ cell tumors remains a valid question. In the TI‐CE regimen, TDM was demonstrated to be feasible in routine practice and really allowed to control target AUC more accurately compared to previous reports, avoiding both underexposure and overexposure to carboplatin. In our study, the rate of CR observed in this population with very poor prognosis was 44.3% and increased to 69.6% of favorable responses. Based on our study and if the benefit of HDCT is proven in the TIGER trial, we suggest that the use of carboplatin TDM for dose individualization in current practice should be considered.

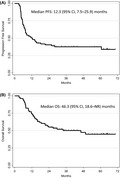

## 1 INTRODUCTION

After first‐line standard chemotherapy, 20% of patients with advanced germ cell tumors (GCT) are not cured.^1^ High‐dose chemotherapy (HDCT) has become the standard of care in relapsed/refractory GCT, based on phase II trials and results of retrospective matched‐pair analyses. This approach has been evaluated since the late 1990 s by different authors in various settings with promising results.^2^, ^3^, ^4^, ^5^, ^6^, ^7^, ^8^ Initially, the administration of high‐dose carboplatin was calculated according to body surface area.^2^, ^9^ In the study of Motzer et al published in 2000, carboplatin was dose escalated by target area under the curve (AUC) among patient cohorts.^6^ This study demonstrated the efficacy of the TI‐CE HDCT plus peripheral blood‐derived stem cell (PBSC) rescue. The regimen associated two rapid recycling dose‐dense regimen of paclitaxel (T) plus ifosfamide (I) followed by three cycles of high‐dose carboplatin (C) and etoposide (E) where the target carboplatin AUC ranged among cohorts from 12 to 32 mg.min/mL. However, the pharmacokinetic study showed that carboplatin AUC measured in serum was lower than target AUC.^6^ It was hypothesized that the glomerular filtration rate measured with plasma clearance of 99MTc‐DTPA used in the dosing formula was underestimated.^6^ In a second trial, the dose of carboplatin was based on the Calvert formula for predicting glomerular filtration rates at higher target AUCs (24 mg.min/mL).^7^ However, the variability among individuals remained large. With this approach, the complete response (CR) rate was 55%.^7^

Underexposure to high‐dose carboplatin leads to lower efficacy of the rescue therapy and overexposure to adverse events such as ototoxicity.^10^ For a better control of patient exposition to carboplatin (evaluated with AUC), we proposed the use of therapeutic drug monitoring (TDM) based on individual ultrafilterable plasma carboplatin measurements. In the present phase II multicenter study, we hypothesized that efficacy and safety of high‐dose TI‐CE could be optimized by this approach. To obtain an AUC target equal to 24 mg.min/mL over 3 days, the dose of carboplatin on day 3 was adapted according to ultrafilterable plasma concentration of carboplatin obtained after the initial dose on day 1. The pharmacokinetic results of this study have been recently published^11^ and the present article reports the clinical results.

## 2 PATIENTS AND METHODS

### 2.1 Study design

This was a multicentric prospective national phase II trial performed by the French Genitourinary Group (GETUG). The objective of the study was to assess the efficacy of the TI‐CE HDCT with optimization of the carboplatin dosage using TDM in patients with relapsed poor‐prognosis GCT.

The treatment consisted in two regimens with paclitaxel plus ifosfamide cycles followed by three high‐dose cycles with carboplatin plus etoposide associated with PBSC support.

Carboplatin doses were individually adjusted at each high‐dose cycle to take into account interindividual variability. The original TDM approach and the results of the pharmacokinetic results have been previously described in details.^11^

### 2.2 Patients

The main inclusion criteria were: age ≥18 years; GCT whatever the histology type (seminomatous or non‐seminomatous), gonadal or extragonadal (retroperitoneal or mediastinal) origin, confirmed by histology and/or tumor markers; relapse after first‐line chemotherapy (either progression after achievement of a clinical partial response or stable disease, progression of markers within 4 weeks after last chemotherapy cycle, and progression on first‐line treatment without achieving at least stable disease or primitive mediastinal origin); and relapse of seminomatous or non‐seminomatous GCT after two lines of treatment (if applicable). Disease progression had to be documented with tumor markers (AFP and/or hCG) and/or by a biopsy. The other inclusion criteria were a performance status score ≤2; biological parameters and physiological functions compatible with administration of HDCT; and absence of prior treatment intensification. The main exclusion criteria were: primitive brain GCT; lesions of growing teratoma; and symptomatic brain metastases despite corticotherapy.

### 2.3 Treatments and carboplatin dose adjustment

The treatment consisted of two cycles (14 days apart) of paclitaxel (200 mg/m^2^, day 1 over 24 h) plus ifosfamide (2 g/m^2^/day from day 2 to day 4) and mesna protection. Leukapheresis for PBSC collection started at day 11 to obtain 9 × 10^6^ CD34+ cells/kg. These two cycles of paclitaxel plus ifosfamide were followed by three cycles (14‐day to 21‐day intervals) of high‐dose carboplatin (total AUC of 24 mg.min/mL over 3 days) and etoposide (400 mg/m^2^/day) given for three consecutive days in each cycle with PBSC support.

In each cycle, carboplatin was administered as a daily 1‐h infusion in 5% dextrose for three consecutive days. The initial carboplatin dose (day 1 and day 2 of high‐dose cycle 1) was calculated as follow: 8 × CLp, where 8 is the daily target AUC and CLp is the predicted carboplatin clearance (CL) calculated with a previously published equation^12^:

CLp (mL/min) = 110 × (serum creatinine/75)^−0,654^ × (body weight/65)^0,625^ × (age/56)^−0,507^, with serum creatinine in µmol/L, body weight in kg, and age in years.

To limit the risk of overdosing, a value of predicted CL of 200 mL/min was set as the upper limit. Consequently, the maximum daily dose to be administered on day 1 and day 2 of first high‐dose cycle was 1600 mg.

For each carboplatin administration, three blood samples were collected at 5 minutes before the end of infusion, and 1 and 4 h after the end of infusion. After immediate centrifugation of the blood samples, 1 mL of plasma was taken and then ultrafiltered using the Amicon MPS1 micropartition system with YM‐T membrane. Carboplatin levels in the plasma ultrafiltrate obtained at day 1 of each cycle were measured by means of flameless atomic absorption spectrophotometric analysis.^13^ A pharmacokinetic analysis based on a Bayesian approach allowed obtaining individual CL on C1D1 for each patient, as previously described.^11^

The carboplatin dose of day 3 of high‐dose cycle 1 was adjusted to obtain a total target AUC of 24 mg.min/mL based on the hypothesis that CL was constant over the 3 days of the cycle:

Dose_D3_ (mg) = [24 – (Dose_D1_ + Dose_D2_)/actual CL_D1_] × actual CL_D1_

For the subsequent cycles of treatment, the AUC target remained 24 mg.min/mL if no major ototoxicity was observed. Otherwise, the target AUC was reduced to 18 mg.min/mL. If serum creatinine did not change, the first dose was calculated using the actual CL of day 1 of the preceding cycle and the same procedure of dose adaptation was conducted on day 3 based on the analysis of day 1 concentrations. If serum creatinine had changed, then the predicted CL was recalculated with the updated value of serum creatinine.

### 2.4 Statistical analysis

The primary endpoint was the CR rate after chemotherapy with or without surgery. The main secondary endpoints were progression‐free survival (PFS), overall survival (OS), toxicity evaluated with NCI‐CTC v3.0 (except for ototoxicity which was evaluated by audiogram parameters), and evaluation of carboplatin TDM performance to achieve AUC 24 mg.min/mL.

For ototoxicity assessment, audiograms were planned before and after two HDCT cycles with pure‐tone audiometry at frequency levels ranging from 250 to 8000 Hz. Overall hearing loss was assessed by calculating Pure‐Tone Average (PTA), which refers to the average of hearing threshold levels at the following frequencies: 500, 1000, 2000, and 4000 Hz. Because platinum treatment mainly affects the high frequencies, the decibel thresholds measured at 4000 and 8000 Hz for both ears were averaged to obtain a mean threshold at 4000–8000 Hz (m4000–8000) that would be more indicative of a specific effect of platinum. Then, the International American Speech‐Language‐Hearing Association (ASHA) criteria that defined hearing loss as a hearing threshold that exceeded 20 dB were used to qualify the PTA4 and m4000‐8000 as follows: mild: 21 to 40 dB; moderate: 41 to 55 dB; moderately severe: 56 to 70 dB; severe: 71 to 90 dB; and profound: >90 dB.^14^, ^15^

The primary analysis was performed on the modified intent‐to‐treat population (mITT) defined as patients who received at least two cycles of HDCT and evaluable for efficacy. Safety population was defined as patients who received at least one dose of treatment. Simon's two‐stage minimax design^16^ was used to test the null hypothesis that the true CR was ≤50% (considered unacceptable),^7^ as opposed to the alternative hypothesis of ≥65% (considered promising). Assuming a type I error of 5% and a power of 90%, 29 or more CR were required among the first 57 patients to proceed to the second stage, whereby an additional 36 patients would be enrolled, for a total of 93 patients. If 55 or more patients achieved a CR at the end of the second stage, this regimen would be declared promising in this patient population.

## 3 RESULTS

### 3.1 Patient disposition and characteristics at inclusion

**FIGURE 1:**
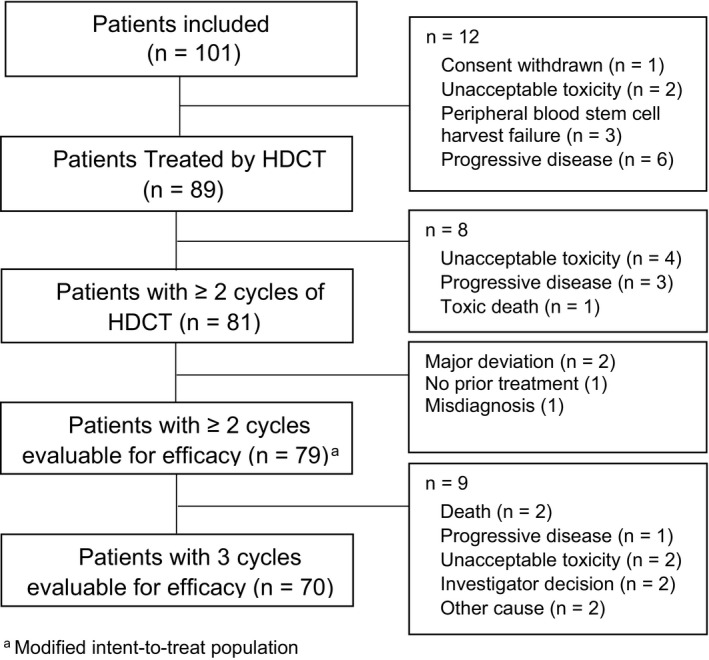
Study flow chart.

A total of 101 patients were enrolled in eight French centers from March 2009 to November 2015, and 81 patients received ≥2 HDCT cycles (12 patients were not treated by HDCT and 8 did not receive ≥2 cycles). Two patients had a major deviation to protocol. Therefore, the mITT population included 79 patients (Figure 1). Seventy patients received the three cycles of HDCT. Reasons for treatment discontinuation of patients enrolled in the study are detailed in Figure 1.

Characteristics of patients at inclusion are shown in Table 1. Some disease characteristics were associated with poor prognosis: 20 patients had mediastinal primary tumors (all were non‐seminomatous), 37 patients had at least three metastatic sites, and 54 patients had hCG level ≥1000 IU/L. Moreover, 41 patients received ≥2 prior lines of metastatic chemotherapy and 23 patients progressed within 4 weeks. A total of 72% of the 59 patients who received one line of metastatic chemotherapy were classified in the high‐ (n = 19) and very high‐risk groups (n = 17) according to the International Prognostic Score.^17^

**TABLE 1:** Patient characteristics at inclusion.

Characteristics	N = 101
Age (years), median (range)	34 (19–57)
Histology, n (%)	
Non‐seminoma	81 (81)
Seminoma	19 (19)
Missing	1
Primary tumor site, n (%)	
Testis	71 (71)
Retroperitoneal	10 (10)
Mediastinal	20 (20)
≥3 metastatic sites	37 (37)
Main metastatic sites, n (%)	
Lung	57 (57)
Lymph node	55 (55)
Liver	35 (35)
Bone	11 (11)
Brain	11 (11)
Serum hCG (IU/L)	
Median	2322 [0; 63526]
≥1000, n (%)	54 (57)
Missing	7
Serum AFP (ng/mL)	
Median	8 [.1; 57658]
≥1000, n (%)	13 (13)
Missing	4
Number of previous metastatic chemotherapy lines, n (%)	N=96^b^
0	1 (1)
1	59 (61)
2	26 (26)
3	15 (15)
Initial response to first‐line metastatic chemotherapy, n (%)	N = 96
CR or PR with negative tumor markers	33 (35)
PR with positive markers or SD	41 (43)
PD	21 (22)
Missing	1
Progression‐free interval to first‐line metastatic chemotherapy, n (%)	N = 96
<4 weeks (platinum refractory)	23 (24)
4–12 weeks	25 (26)
International Prognostic Score, n (%)	N = 59^a^
Low	6 (10)
Intermediate	10 (19)
High	19 (39)
Very high	17 (33)
Missing	7

Abbreviations: AFP, alpha fetoprotein; CR; complete response; hCG, human chorionic gonadotropin; PD, progressive disease; PR, partial response; SD, stable disease.

^a^ Patients with one line of metastatic chemotherapy.

^b^ 90 patients received BEP as first line.

### 3.2 Clinical response

In the mITT population (n = 79), CR rate was 44.3% (n = 35; 95% CI: 33.1–55.6), including 13 patients with CR after chemotherapy alone and 22 after chemotherapy plus surgery (Table 2). In addition, 20 patients (25.3%) achieved PR with negative tumor markers. Therefore, the total favorable response rate was 69.6% (95% CI: 58.2–79.5). For patients who received three HDCT cycles, the favorable response rate was 72.9%.

**TABLE 2:** Efficacy results of induction treatment and high‐dose chemotherapy.

	≥ 2 HDCT cycles (N = 79) ^a^	≥ 3 HDCT cycles (N = 70)
Treatment response		
cCR	13 (16.5)	13 (18.6)
pCR	16 (20.3)	14 (20.0)
sCR	6 (7.6)	5 (7.1)
PRm−	20 (25.3)	19 (27.1)
PRm+	8 (10.1)	8 (11.4)
SD	3 (3.8)	3 (4.3)
PD	11 (13.8)	8 (11.4)
Not evaluable	2 (2.5)	0
Study endpoints		
CR	35 (44.3)	32 (45.7)
CR or PRm−	55 (69.6)	51 (72.9)

Abbreviations: cCR, clinical complete response; pCR, pathologic complete response; sCR, surgical complete response; HDCT, high‐dose chemotherapy; PD, progressive disease; PRm–, partial response with negative tumor markers; PRm+, partial response with positive tumor markers; SD, stable disease.

Results are presented as n (%).

^a^ Modified intent‐to‐treat population.

**FIGURE 2:**
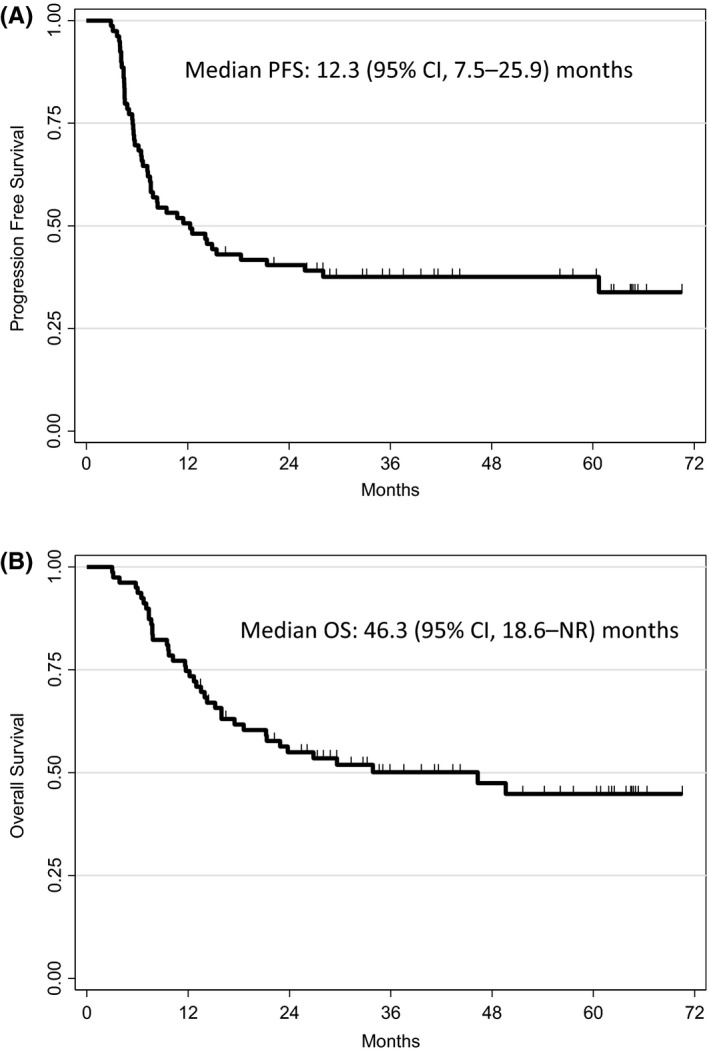
Progression‐free survival (PFS) (A) and overall survival (OS) in modified intent‐to‐treat population (n = 79).

Progression or death was observed in 50 patients (63%) after a median follow‐up of 44 months. The median PFS for the overall population was 12.3 (95% CI: 7.5–25.9) months and was not reached for patients with favorable response (Table 3 and Figure 2A). The 2‐year PFS rate was 40.4% for the overall mITT population and 56.3% for patients with favorable response. For patients with high‐ and very high‐risk at first relapse or those treated with ≥2nd line chemotherapy (n = 51 mITT), the 2‐year PFS rate was 41.1% (Table 3).

**TABLE 3:** Survival of patients in modified intent‐to‐treat population (n = 79) and in subgroups of interest (median follow‐up, 44 months).

	N	Survival			
		Median (months)	95% CI	24 months (%)	36 months (%)
Progression‐free survival					
≥2 HDCT cycles^a^	79	12.3	7.5–25.9	40.4	37.6
≥3 HDCT cycles	70	12.5	7.6–28.1	41.3	38.2
CR or PR with negative tumor markers	55	NR	‐	56.3	52.1
High/very high‐risk^b^ or ≥2nd line (≥2 HDCT cycles)	51	8.4	6.5–NR	41.1	38.8
Overall survival					
≥2 HDCT cycles ^a^	79	46.3	18.6–NR	55.0	50.1
≥3 HDCT cycles	70	46.3	18.6–NR	56.3	50.9
CR or PR with negative tumor markers	55	NR	‐	70.6	63.8
High/very high‐risk ^b^ or ≥2nd line (≥2 HDCT cycles)	51	26.8	12.9–NR	50.2	47.9

Abbreviations: CR, complete response; HDCT, high‐dose chemotherapy; NR, not reached; PR, partial response.

^a^ Modified intent‐to‐treat population

^b^ International prognosis score for patients with one line of metastatic chemotherapy.

During the follow‐up, median OS was 46.3 months (95% CI: 18.6–not reached) for the mITT population and was not reached for patients with favorable response (Table 3 and Figure 2B). The 2‐year OS rate was 55.0% for the mITT population and 70.6% for patients with favorable response. For patients with high and very high‐risk with one line of metastatic chemotherapy or treated with ≥2nd line chemotherapy (n = 51), the 2‐year OS rate was 50.2% (Table 3).

### 3.3 Safety

Hematologic toxicity was the major toxicity encountered as expected in this setting. One toxic death due to sepsis was reported during the study. The other Grade 3–4 non‐hematological toxicities were nausea (n = 18), diarrhea (n = 16), pain (n = 13), vomiting (n = 11), fatigue (n = 11), hypokalemia (n = 9), fever without neutropenia (n = 4), mucositis/stomatitis (n = 3), and peripheral neuropathy (n = 3).

Audiograms were available for 36 patients before HDCT cycles (pre‐HDCT) and for 47 patients with ≥2 HDCT cycles (post‐HDCT). Among these 47 post‐HDCT audiograms, 12 were obtained after three HDCT cycles instead of two.

**FIGURE 3:**
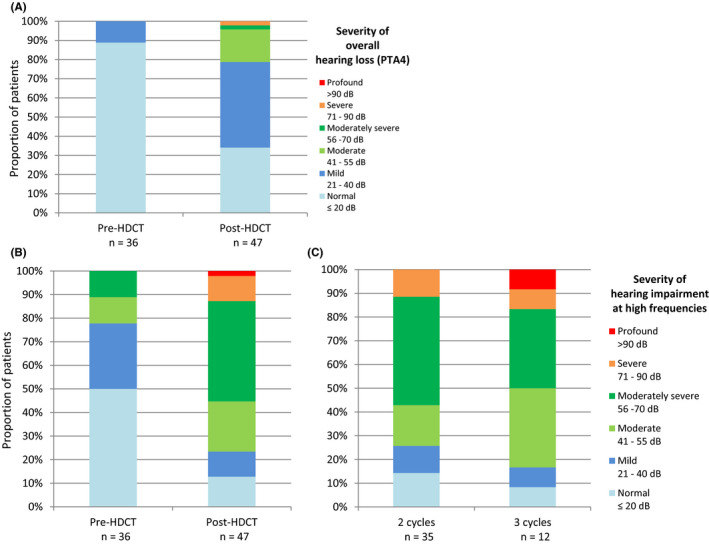
Overall hearing impairment (measured with PTA) before and after completion of HDCT (A); High frequencies impairment (measured with m4000‐8000) before and after completion of HDCT (B) and detail for patients who received 2 cycles *vs*. 3 cycles of HDCT (C).

The results presented in Figure 3 show that HDCT increased the severity of ear impairment, especially at high frequencies (Figure 3B and 3C). Among the patients presented in the Figure 3A, only 33 had both before and after treatment ear evaluation and the analysis restricted to these 33 patients showed that ear impairment appears in the same proportion as when all patients are analyzed together (45% had mild, 21 had moderate, 3% had moderately severe, and 3% had severe overall hearing loss based on PTA).

Concerning renal toxicity, there was a trend for a decrease of CL over the three cycles as a nephrotoxic effect of high doses of carboplatin. The median decrease (min‐max) of CL was −7.7% (−41.9%; +19.9%) between cycles 1 and 2 (by comparing the first day of each cycle), +3% (−29.4%; +27.9%) between cycles 2 and 3, and −11.6% (−36.1%; +19.5%) between cycle 1 and cycle 3.

## 4 DISCUSSION

In this phase II trial of TI‐CE HDCT for relapsed GCT, 35 (44.3%) patients achieved CR and 20 (25.3%) additional patients achieved PR with negative tumor markers. With a median follow‐up of 44 months, median PFS and OS were 12.3 (95% CI, 7.5–25.9) and 46.3 (95% CI, 18.6–not reached) months, respectively.

Although the primary objective (CR ≥65%) was not achieved, these results raise several questions. Thus, the classical risk factors predicted a poor outcome for the GCT patients included in the cohort.^18^ Indeed, 20% of patients had mediastinal primary tumors (all non‐seminomatous), 37% had ≥3 metastatic sites, metastatic sites associated with poor prognosis were frequent (liver, 35%; bone, 11%; brain, 11%), 57% of patients had hCG ≥1000 IU/L, 41% received ≥2 previous lines of metastatic chemotherapy, only 35% presented a favorable response to first‐line treatment, 24% were platinum refractory (disease progression within 4 weeks after cisplatin‐based chemotherapy), and 26% progressed within 3 months to first line, 72% of the 59 patients with one line of metastatic chemotherapy were at high‐ and very high‐risk according to the International Prognostic Score. Therefore, the prognosis of these patients was poorer compared to previous GCT cohorts treated with TI‐CE HDCT regimen.^6^, ^7^, ^19^

It is also important to underline that the value of the CR rate for null hypothesis was set at 50% according to the results from the study of Kondagunta et al (CR rate, 48.9%; 95% CI, 34.1–63.9), which evaluated a population with a better prognosis.^7^ The goal of the present study was to improve this rate by using individual carboplatin dosing. It was anticipated that the design with TDM of carboplatin would be promising if 65% of patients achieved CR. Although our results did not meet our too ambitious pre‐specified hypothesis in a population with poor prognosis, 20 additional patients achieved PR with negative tumor markers, thus giving a total rate of favorable responses equal to 69.6%. Furthermore, for the subgroup of patients with high‐ and very high‐risk treated with one line of metastatic chemotherapy (or treated with ≥2nd line), the 2‐year PFS rate was 41.1%. These latter results compare favorably with those published by Lorch et al^17^ in a retrospective study of 1,984 patients with relapsed metastatic GCT after a first line of treatment where PFS at 2 years was 33% and 22% for patients at high and very high‐risk, respectively.

The originality of our approach was the individualization of carboplatin dose for achieving the target AUC (24 mg.min/mL) based on pharmacokinetic data. The performance of this PK‐based individualization method has been previously published by Moeung S, et al^11^ and demonstrated that carboplatin AUC was better controlled in comparison with previous studies.^6^, ^7^ In cycle 1, TDM resulted in an absolute change of the total dose over 3 days >20% for 20 of 89 patients (from −33% to +44%).^11^In cycle 2 and cycle 3, 23 of 80 patients and 22 of 72 patients had an absolute change of dose >20% (from −42% to +30% and from −40% to +24%), respectively. As a consequence of these dose changes, the mean actual AUC was 24.4 mg.min/mL per cycle with 10th and 90th percentiles equal to 22.4 and 26.8 mg.min/mL, respectively, thus demonstrating both the precision for targeting AUC and the low inter‐patient variability.

Although we showed that the implementation of such a TDM procedure was feasible at a multicenter level (eight participating centers to the current study), we should acknowledge that the procedure may be difficult to organize in some institutions. It required that the samples are centrifuged on site with specific ultrafiltration systems and then transmitted to a pharmacology or toxicology laboratory for the samples to be analyzed on day 1 or day 2. Afterwards, the pharmacokinetic analysis had to be done by an expert team to calculate the dose to be given at day 3.

The analysis of the adverse events observed in our study indicates that the choice of 24 mg.min/mL per cycle as a target for high‐dose carboplatin proposed by Kondagunta et al.^7^ was relevant. Besides the expected hematologic toxicity, irreversible ototoxicity and nephrotoxicity are frequently reported with high doses. Ototoxicity is reported even in patients who did not present clinical hearing impairment before the high‐dose regimen..^10^, ^20^ In our cohort, 10% of patients had an overall hearing loss >20 dB before starting HDCT cycles which increased at 65% after HDCT. When focusing on ear impairment affecting high frequencies, 50% of patients had a high frequencies hearing loss >20 dB before starting HDCT cycles, mainly as a consequence of previous cisplatin treatment. This proportion of patients rose to 87% after HDCT with six patients experiencing severe or profound hearing loss at high frequencies. Ototoxicity has not been frequently and uniformly described in previous TI‐CE trials. Nevertheless, as the known correlation between ototoxicity and cumulative exposure of carboplatin, it can be hypothesized that avoiding overexposure with the TDM‐individualized dosing approach helps to limit the occurrence of severe ototoxicity. In the same way, concerning nephrotoxicity, we observed a slight drop in CL over the three cycles, likely due to the nephrotoxicity of the high‐dose regimen carboplatin suggesting that higher target AUC would have been associated with unacceptable nephrotoxicity.

Despite the absence of a prospective control group, which is the main limitation of our study, these findings strongly support the value of TDM of carboplatin by avoiding over‐exposition.

Indeed, the results clearly show that this method is not only feasible, but lead to a lower inter‐patient variability of carboplatin AUC than alternative methods to predict CL. This is especially noteworthy in the setting of a multicentric study, by contrast to the previous monocentric studies.^6^, ^7^

In conclusion, the rate of CR observed in this population with very poor prognosis was 44.3% and increased to 69.6% of favorable responses. TDM was demonstrated to be feasible in routine practice and really allowed to control target AUC more accurately compared to previous reports, avoiding both underexposure and overexposure to carboplatin. The superiority of HDCT over standard chemotherapy as initial salvage treatment of patients with relapsed or refractory GCT is being assessed in the ongoing TIGER trial NCT02375204. Based on our study and if the benefit of HDCT is proven in the TIGER trial, we suggest that the use of carboplatin TDM for dose individualization in current practice should be considered.

## CONFLICTS OF INTEREST

The authors declare no potential conflict of interest related to this study.

## ETHICAL STATEMENT

This study was approved by the regional Ethics Committee (“Comité de Protection des Patients CPP Sud Ouest et Outre Mer I”) and the French Health Products Safety Agency. Written informed consent was obtained from each patient. This study was registered in ClinicalTrial.gov (NCT00864318).

## Data Availability

The data that support the findings of this study are available from the corresponding author upon reasonable request.
